# Clinical characteristics, antimicrobial resistance and capsular types of community-acquired, healthcare-associated, and nosocomial *Klebsiella pneumoniae* bacteremia

**DOI:** 10.1186/s13756-018-0426-x

**Published:** 2019-01-03

**Authors:** Chih-Han Juan, Chien Chuang, Chi-Han Chen, Lo Li, Yi-Tsung Lin

**Affiliations:** 1 Division of Infectious Diseases, Department of Medicine, Taipei Veterans General Hospital, Number 201, Section 2, Shih-Pai Road, Beitou District, Taipei, 11217 Taiwan; 2 Department of Medicine, School of Medicine, National Yang-Ming University, Taipei, Taiwan; 3 Institute of Emergency and Critical Care Medicine, National Yang-Ming University, Taipei, Taiwan

**Keywords:** *Klebsiella pneumoniae*, Bacteremia, Community-acquired, Healthcare-associated, Nosocomial, Antimicrobial resistance, Capsular type

## Abstract

**Background:**

*Klebsiella pneumoniae* bacteremia is a major cause of morbidity and mortality worldwide. We aimed to compare the clinical characteristics, distribution of capsular types, and antimicrobial resistance of *K. pneumoniae* bacteremia among community-acquired (CA), healthcare-associated (HCA), and nosocomial infections.

**Methods:**

This retrospective study of patients with *K. pneumoniae* bacteremia was conducted at Taipei Veterans General Hospital from January to December 2015. Clinical characteristics of *K. pneumoniae* bacteremia were collected. The *K. pneumoniae* isolates were subjected to antimicrobial susceptibility testing and capsular genotyping.

**Results:**

In total, 337 patients with *K. pneumoniae* bacteremia were identified: 70 (20.8%), 102 (30.3%), and 165 (48.9%) presented with CA, HCA, and nosocomial infection, respectively. The 28-day mortality of HCA bacteremia was lower than that of nosocomial bacteremia (17.6% versus 30.9%, *p* = 0.016); however, that of the HCA and CA bacteremia was similar (17.6% versus 14.3%, *p* = 0.557). CA isolates had the highest prevalence of virulent capsular types (51.4%), followed by HCA (36.3%) and nosocomial isolates (19.4%). The proportion of multidrug-resistant (MDR) isolates was highest in nosocomial infections (41.8%), followed by HCA (23.5%) and CA infections (5.7%).

**Conclusion:**

CA, HCA and nosocomial *K. pneumoniae* are distinct entities, as evidenced by the differences in clinical characteristics, antimicrobial resistance, and capsular types found in this study.

## Background

*Klebsiella pneumoniae* bacteremia is a major cause of morbidity and mortality worldwide [1]. It is the second leading cause of gram-negative bacteremia, and its case fatality rate was 20% in a population-based study in Canada [2]. Community-acquired (CA) invasive syndromes encompass a variety of clinical presentations associated with *K. pneumoniae* bacteremia, including liver abscess and distant septic metastasis, which are seen more commonly in Taiwan and the Asian companies in comparison with Western countries. However, these syndromes were rarely reported in western countries [3]. Nosocomial *K. pneumoniae* bacteremia has been associated with a higher mortality rate than CA bacteremia [4–6], and these isolates are known for harboring resistance genes encoding expanded-spectrum β-lactams or carbapenems [7], which is less reported in CA isolates.

The presence of polysaccharide capsule is one of the most important virulence factors associated with *K. pneumoniae* [8]. Capsular types K1 and K2 are the most frequently observed virulent types and are usually associated with community-onset pyogenic infections in Asian countries [9]. Other capsular types, such as K5, K20, K54, and K57, were recently identified in individuals with community-onset pyogenic *K. pneumoniae* infections in Asian countries [10]. The capsular type K1 isolates were more common in community-onset infection than in nosocomial infection, whereas non-typeable isolates were more common in nosocomial infection than in community-onset infection in a previous study conducted in Taiwan [4]. *rmpA*, a regulator of the mucoid phenotype, and a gene known as an extracapsular polysaccharide synthesis regulator, can positively control the mucoid phenotype of *K. pneumoniae*, and it is also considered as an important virulence factor [11].

Community-onset bacteremia is classified into healthcare-associated (HCA) infections for patients who recently received healthcare services and underwent medical procedures, and CA infections for the remaining patients [12] because HCA infections were more similar to nosocomial infections in terms of clinical features and antimicrobial resistance [12]. The clinical characteristic and distribution of the capsular type of HCA *K. pneumoniae* bacteremia have been rarely reported in the literature [13–15]. Moreover, the capsular types of *K. pneumoniae* isolates among CA, HCA, and nosocomial bacteremia have never been compared.

Thus, this study aimed to compare the clinical characteristics, antimicrobial resistance, and distribution of the capsular types among CA, HCA, and nosocomial *K. pneumoniae* bacteremia. Moreover, the question of whether HCA bacteremia is a category distinct from CA and nosocomial bacteremia was also addressed.

## Methods

### Study design and population

This descriptive, retrospective study of consecutive patients with *K. pneumoniae* bacteremia was conducted at Taipei Veterans General Hospital, a 2900-bed tertiary-care teaching hospital in Taiwan, from January to December 2015. The study protocol was approved by the institutional review board of Taipei Veterans General Hospital.

The medical records of patients with positive blood culture for *K. pneumoniae* were reviewed, and their clinical information was obtained. CA *K. pneumoniae* bacteremia is defined as *K. pneumoniae*-positive isolates identified in patients upon admission or within 48 h of admission who did not fit the criteria for HCA bacteremia. HCA *K. pneumoniae* bacteremia is defined as *K. pneumoniae*-positive isolates identified in patients upon admission or within 48 h of admission meeting any of the following criteria [12]: having received intravenous therapy at home or in an outpatient clinic within the last 30 days; having received renal dialysis in a hospital or clinic within the last 30 days; having been hospitalized for 2 or more days within the last 90 days; or having resided in a nursing home or long-term care facility. Nosocomial *K. pneumoniae* bacteremia is defined as *K. pneumoniae*-positive isolates identified in patients more than 48 h after admission. The first blood culture of patients with two or more positive blood cultures was included, for the duration of their hospital admission. Patients < 20 years of age and those with incomplete medical records were excluded.

### Data collection

We collected clinical information on the demographic characteristics of the patients, location at the time of culture, source of bacteremia, co-morbidities, immunosuppression, surgeries, invasive procedures or devices, surgical drainage, mechanical ventilation, antimicrobial therapy, severity of illness, outcome, and mortality. Appropriate empirical antimicrobial therapy is defined as the administration of at least one antimicrobial agent to which the causative pathogen is susceptible within 24 h of the onset of clinical sepsis at the approved route and dosage for the affected target organ(s). Appropriate definite antimicrobial therapy is defined as the administration of at least one antimicrobial agent to which the causative pathogen is susceptible after obtaining the result of the antimicrobial susceptibility test within 24 h at the approved route and dosage for the affected target organ(s). Prior antibiotic exposure is defined as at least 2 days of therapy within 30 days prior to acquiring bacteremia. We used the Pitt bacteremia score and the Acute Physiology and Chronic and Prevention Evaluation (APACHE) II score to determine the severity of illness within 24 h of the onset of bacteremia [16, 17].

### Microbiological studies

All *K. pneumoniae* isolates were identified using matrix-assisted laser desorption-ionisation time-of-flight mass spectrometry (bioMérieux SA, Marcy l’Etoile, France). Antimicrobial susceptibility to this bacterium was determined using the VITEK2 system (bioMérieux, Marcy l’Etoile, France) and interpreted according to the guidelines of the Clinical and Laboratory Standards Institute 2017 [18]. Multidrug-resistant (MDR) *K. pneumoniae* isolate is defined as non-susceptibility to at least one agent in three or more antimicrobial categories [19].

To detect the capsular genotypes of *K .pneumoniae* isolates, we performed *cps* genotyping using the polymerase chain reaction of K-serotype-specific alleles at the *wzy* loci, including serotypes K1, K2, K5, K20, K54, and K57 [10]. The detection of *rmpA* and *rmpA2* genes was performed as described previously [20].

### Statistical analyses

Categorical variables were compared using the chi-square or Fisher’s exact test. The analyses of continuous variables were conducted using the Student’s t test and Mann–Whitney U test (Wilcoxon rank-sum test). A two-tailed *p* value < 0.05 was considered statistically significant. All statistical analyses were performed using the Statistical Package for the Social Sciences software, version 17.0 (SPSS Inc., Chicago, IL, the USA).

## Results

### Clinical characteristics of the patients with CA, HCA, and nosocomial *K. pneumoniae* bacteremia

In total, 339 consecutive patients with *K. pneumoniae* bacteremia were identified and 2 (0.6%) patients were excluded due to missing data of treatment. Of which, 70 (20.8%), 102 (30.3%), and 165 (48.9%) presented with CA, HCA, and nosocomial infections, respectively. The mean age of the patients was 69.9 ± 15.3 years, and there was male predominance (*n* = 202, 59.9%). Among them, 67 (19.9%) had polymicrobial bacteremia. The crude 28-day mortality rate was 23.4%.

The clinical characteristics of the patients with CA, HCA, and nosocomial *K. pneumoniae* bacteremia are shown in Table 1. With regard to infection source, the prevalence of pneumonia (19.6% versus 5.7%, *p* = 0.013) and primary bacteremia (20.6% versus 7.1%, *p* = 0.016) was significantly higher in the HCA group than in the CA group, and that of urinary tract infection was also significantly higher in the HCA group than in the nosocomial group (17.6% versus 9.1%, *p* = 0.039). The prevalence of intraabdominal infection was higher in the CA group than in the nosocomial group (37.1% versus 21.8%, *p* = 0.015). The prevalence of liver abscess as a primary site of infection was similar between the HCA and nosocomial groups (4.9% versus 1.2%, *p* = 0.110), but it was lower in the HCA group than in the CA group (4.9% versus 20.0%, *p* = 0.002).

**Table 1:** Clinical characteristics of patients with community-acquired (CA), healthcare-associated (HCA), and nosocomial *K. pneumoniae* bacteremia

Variable	CA (*n* = 70)	HCA (*n* = 102)	Nosocomial (*n* = 165)	*p* value		
				CA vs. HCA	HCA vs. Nosocomial	CA vs. Nosocomial
Demographics						
Age, (Mean ± SD), years	71.43 ± 13.64	72.37 ± 14.39	67.71 ± 16.32	0.696	0.031	0.122
Gender, male	37 (52.9)	70 (68.6)	95 (57.6)	0.036	0.071	0.505
Days of hospitalization prior to culture, median (IQR), days	0.0 (0.0–0.0)	0.0 (0.0–0.0)	14.0 (6.0–25.0)	N/A	< 0.001	< 0.001
Polymicrobial infection	12 (17.1)	16 (15.7)	40 (24.2)	0.799	0.095	0.231
Primary site of infection						
Pneumonia	4 (5.7)	20 (19.6)	36 (21.8)	0.013	0.666	0.002
Urinary tract	14 (20.0)	18 (17.6)	15 (9.1)	0.697	0.039	0.020
Intra-abdomen^a^	26 (37.1)	32 (31.4)	36 (21.8)	0.432	0.082	0.015
Liver abscess	14 (20.0)	5 (4.9)	2 (1.2)	0.002	0.110	< 0.001
Skin and soft tissue	2 (2.9)	1 (1.0)	4 (2.4)	0.567	0.652	1.000
Intravenous catheter	0 (0.0)	1 (1.0)	8 (4.8)	1.000	0.160	0.109
Primary bacteremia	5 (7.1)	21 (20.6)	60 (36.4)	0.016	0.006	< 0.001
Disseminated infection	4 (5.7)	4 (3.9)	2 (1.2)	0.717	0.206	0.066
Underlying disease						
Malignancy	12 (17.1)	49 (48.0)	95 (57.6)	< 0.001	0.129	< 0.001
Diabetes mellitus	27 (38.6)	41 (40.2)	46 (27.9)	0.830	0.037	0.105
Chronic kidney disease	28 (40.0)	35 (34.3)	57 (34.5)	0.447	0.969	0.426
Hemodialysis	0 (0.0)	5 (4.9)	15 (9.1)	0.081	0.206	0.007
Congestive heart failure	4 (5.7)	13 (12.7)	14 (8.5)	0.193	0.262	0.597
Liver cirrhosis	6 (8.6)	10 (9.8)	14 (8.5)	0.785	0.714	0.983
Cerebral vascular disease	7 (10.0)	15 (14.7)	28 (17.0)	0.364	0.625	0.170
Chronic obstructive lung disease	2 (2.9)	7 (6.9)	6 (3.6)	0.313	0.234	1.000
Collagen vascular disease	2 (2.9)	3 (2.9)	6 (3.6)	1.000	1.000	1.000
Transplantation	1 (1.4)	4 (3.9)	5 (3.0)	0.649	0.735	0.672
Immunosuppression^b^	5 (7.1)	41 (40.2)	69 (41.8)	< 0.001	0.794	< 0.001
Invasive procedures and devices at onset of bacteremia	14 (20.0)	30 (29.4)	98 (59.4)	0.165	< 0.001	< 0.001
Central venous catheter	1 (1.4)	3 (2.9)	45 (27.3)	0.647	< 0.001	< 0.001
Nasogastric/Nasojejunal tube	9 (12.9)	21 (20.6)	72 (43.6)	0.189	< 0.001	< 0.001
Urinary catheter	8 (11.4)	16 (15.7)	53 (32.1)	0.429	0.003	0.001
Endotracheal tube^c^	1 (1.4)	2 (2.0)	24 (14.5)	1.000	< 0.001	0.002
Tracheostomy	1 (1.4)	0 (0.0)	18 (10.9)	0.407	< 0.001	0.016
Surgical drainage	0 (0.0)	0 (0.0)	16 (9.7)	N/A	< 0.001	0.004
Surgery within 2 weeks	0 (0.0)	5 (4.9)	32 (19.4)	0.081	0.001	< 0.001
Prior antibiotic exposure						
Any antibiotic	0 (0.0)	30 (29.4)	109 (66.1)	< 0.001	< 0.001	< 0.001
1st or 2nd generation cephalosporin^d^	0 (0.0)	16 (15.7)	46 (27.9)	< 0.001	0.022	< 0.001
3rd or 4th generation cephalosporin^e^	0 (0.0)	4 (3.9)	25 (15.2)	0.147	0.004	< 0.001
β-lactam and β-lactamase inhibitor^f^	0 (0.0)	11 (10.8)	55 (33.3)	0.003	< 0.001	< 0.001
Carbapenem^g^	0 (0.0)	5 (4.9)	31 (18.8)	0.081	0.001	< 0.001
Fluoroquinolone^h^	0 (0.0)	4 (3.9)	23 (13.9)	0.147	0.011	< 0.001
Aminoglycoside^i^	0 (0.0)	1 (1.0)	9 (5.5)	1.000	0.095	0.061
Tigecycline	0 (0.0)	1 (1.0)	12 (7.3)	1.000	0.020	0.020
Glycopeptide^j^	0 (0.0)	1 (1.0)	20 (12.1)	1.000	0.001	0.001
Metronidazole	0 (0.0)	2 (2.0)	11 (6.7)	0.514	0.140	0.037
Pitt bacteremia score, median (IQR)	1.0 (0.0–2.0)	1.0 (0.0–3.0)	2.0 (0.0–4.0)	0.517	0.054	0.012
APACHE II score, median (IQR)	11.0 (9.0–16.0)	17.0 (11.0–22.0)	19.0 (14.0–24.0)	< 0.001	0.016	< 0.001

Data are presented as number (%) of patients, unless stated otherwise

*SD* standard deviation, *IQR* interquartile range, *APACHE* Acute Physiology And Chronic Health Evaluation, *N/A* not applicable

^a^Intra-abdominal infection was defined as infections of single organs of abdomen with or without extension into the peritoneal space with exclusion of liver abscess

^b^Immunosuppression was defined as meeting one of the following criteria: neutropenia, use of corticosteroids, or receiving chemotherapy

^c^Endotracheal tube was defined as patient being intubated at the onset of bacteremia

^d^Including cefazolin and cefuroxime

^e^Including cefoperazone, ceftriaxone, cefotaxime, cefepime, and cefpirome

^f^Including amoxicillin/clavulanate, ampicillin/sulbactam, piperacillin/tazobactam, and ticarcillin/clavulanate

^g^Including ertapenem, imipenem, meropenem, and doripenem

^h^Including ciprofloxacin, levofloxacin, and moxifloxacin

^i^Including amikacin, gentamicin and isepamicin

^j^Including vancomycin and teicoplanin

With regard to co-morbidities, malignancy and immunosuppression were both observed more frequently in the HCA group and nosocomial group as opposed to the CA group (48.0% versus 17.1%, *p* < 0.001; 40.2% versus 7.1%, *p* < 0.001, respectively). The prevalence of diabetes was higher among patients with HCA bacteremia than those with nosocomial bacteremia (40.2% versus 27.9%, *p* = 0.037). The proportion of performing invasive procedures and using medical devices was similar between the HCA and CA bacteremia groups (29.4% versus 20.0%, *p* = 0.165). However, a higher proportion was observed in the nosocomial bacteremia group than in the HCA bacteremia group (59.4% versus 29.4%, *p* < 0.001).

Prior antibiotic exposure was less common in the HCA group than the nosocomial group (29.4% versus 66.1%, *p* < 0.001). The low prevalence of prior antibiotic exposure was notably in the CA group (0.0%). Patients with HCA bacteremia had a higher disease severity than those with CA bacteremia, as indicated by the median APACHE II score (17.0 versus 11.0, *p* < 0.001), but a lower severity than those with nosocomial bacteremia (17.0 versus 19.0, *p* = 0.016) (Table 1).

### Clinical outcomes of patients with CA, HCA, and nosocomial *K. pneumoniae* bacteremia

The proportion of appropriate empirical antimicrobials in the HCA group was lower than that in the CA group (89.2% versus 97.1%, *p* = 0.077), but higher than that in the nosocomial group (89.2% versus 80.6%, *p* = 0.063) with borderline statistical significance (Table 2).

**Table 2:** Clinical outcomes of patients with community-acquired (CA), healthcare-associated (HCA), and nosocomial *K. pneumoniae* bacteremia

Variable	CA (*n* = 70)	HCA (*n* = 102)	Nosocomial (*n* = 165)	*p* value		
				CA vs. HCA	HCA vs. Nosocomial	CA vs. Nosocomial
Appropriate empirical antimicrobial therapy	68 (97.1)	91 (89.2)	133 (80.6)	0.077	0.063	< 0.001
Appropriate definite antimicrobial therapy	65 (92.9)	93 (91.2)	138 (83.6)	0.692	0.080	0.059
Length of stay after bacteremia, median (IQR), days	14.0 (8.0–23.8)	16.0 (9.8–28.0)	17.0 (7.0–30.5)	0.191	0.685	0.406
Septic shock when bacteremia	15 (21.4)	35 (34.3)	58 (35.2)	0.068	0.889	0.038
Respiratory failure requiring mechanical ventilation	6 (8.6)	14 (13.7)	9 (5.5)	0.300	0.019	0.371
Mortality						
In-hospital mortality	12 (17.1)	25 (24.5)	67 (40.6)	0.248	0.007	< 0.001
Crude 28-day mortality	10 (14.3)	18 (17.6)	51 (30.9)	0.557	0.016	0.008

*IQR* interquartile range, *ICU* intensive care unit

Data are presented as number (%) of patients, unless stated otherwise

The prevalence of respiratory failure requiring mechanical ventilation was higher in the HCA group than in the nosocomial group (13.7% versus 5.5%, *p* = 0.019). The 28-day mortality of the HCA group was significantly lower than that of the nosocomial group (17.6% versus 30.9%, *p* = 0.016), whereas that of the HCA and CA groups was similar (17.6% versus 14.3%, *p* = 0.557). The in-hospital mortality of the three groups had a similar trend.

### Microbiological characteristics of the clinical isolates of CA, HCA, and nosocomial *K. pneumoniae* bacteremia

Table 3 depicts the antimicrobial resistance of *K. pneumoniae* isolates among the three groups. *K. pneumoniae* isolates from HCA bacteremia had a higher proportion of wild-type antibiotic susceptibility (isolates that are susceptible to several classes of antibiotics but ampicillin) than those from nosocomial bacteremia (65.7% versus 47.3%, *p* = 0.003). However, the proportion of wild-type antibiotic susceptibility was lower in the HCA isolates than CA isolates (65.7% versus 85.7%, *p* = 0.003). The proportion of MDR isolates from nosocomial infection was the highest (41.8%), followed by isolates from HCA infection (23.5%) and CA infection (5.7%) (Table 3). Similar trend among the three categories could be identified in isolates resistant to ceftriaxone, ciprofloxacin, levofloxacin, and ertapenem.

**Table 3:** Antimicrobial resistance rates of clinical isolates of community-acquired (CA), healthcare-associated (HCA), and nosocomial *K. pneumoniae* bacteremia

Variable	CA (*n* = 70)	HCA (*n* = 102)	Nosocomial (*n* = 165)	*p* value		
				CA vs. HCA	HCA vs. Nosocomial	CA vs. Nosocomial
Amikacin	0 (0.0)	1 (1.0)	7 (4.2)	1.000	0.160	0.107
Gentamicin	1 (1.4)	15 (14.7)	35 (21.2)	0.003	0.185	< 0.001
Cefuroxime	2 (2.9)	23 (22.5)	67 (40.6)	< 0.001	0.002	< 0.001
Ceftriaxone	1 (1.4)	12 (11.8)	52 (31.5)	0.016	< 0.001	< 0.001
Cefepime	0 (0.0)	6 (5.9)	24 (14.5)	0.082	0.029	< 0.001
Ciprofloxacin	1 (1.4)	11 (10.8)	49 (29.7)	0.029	< 0.001	< 0.001
Levofloxacin	1 (1.4)	11 (10.8)	48 (29.1)	0.029	< 0.001	< 0.001
Ertapenem	0 (0.0)	7 (6.9)	27 (16.4)	0.042	0.024	< 0.001
Imipenem	0 (0.0)	1 (1.0)	14 (8.5)	1.000	0.011	0.012
Tigecycline	2 (2.9)	3 (2.9)	5 (3.0)	1.000	1.000	1.000
Trimethoprim-sulfamethoxazole	5 (7.1)	25 (24.5)	61 (37.0)	0.003	0.034	< 0.001
Wild-type antibiotic susceptibility^a^	60 (85.7)	67 (65.7)	78 (47.3)	0.003	0.003	< 0.001
Multidrug resistance^b^	4 (5.7)	24 (23.5)	69 (41.8)	0.002	0.002	< 0.001

Data are presented as number (%) of isolates resistant to the antibiotic indicated, unless stated otherwise

^a^Wild-type antibiotic susceptibility was defined in the isolates as susceptibility to all antibiotics except for ampicillin

^b^Multidrug resistance was defined in the isolates as nonsusceptibility to at least one agent in three or more antimicrobial categories

The distribution of capsular types and the presence of *rmpA*/*rmpA2* genes among all *K. pneumoniae* isolates are shown in Table 4. Of the 337 isolates of *K. pneumoniae* bacteremia, 105 (31.2%) belonged to the six virulent capsular types (K1, K2, K5, K20, K54, and K57), which was more common in the HCA isolates than in nosocomial isolates (36.3% versus 19.4%, *p* = 0.002) but less common in the HCA than in CA isolates (36.3% versus 51.4%, *p* = 0.048). The distribution of capsular type K1/K2 among the three groups had a similar trend. The rate of *rmpA* and *rmpA2* genes was higher in the HCA isolates than in the nosocomial isolates (38.2% versus 21.8%, *p* = 0.004, 36.3% versus 21.2%, *p* = 0.007, respectively), but lower in the CA isolates (38.2% versus 60.0%, *p* = 0.005, 36.3% versus 58.6%, *p* = 0.004, respectively). Moreover, several MDR isolates (*n* = 12, 3.5%) also belonged to the virulent capsular types and they were similarly distributed among the three categories [*n* = 2 (2.9%) in the CA bacteremia group, *n* = 3 (2.9%) in the HCA bacteremia group, and *n* = 7 (4.2%) in the nosocomial bacteremia group].

**Table 4:** Comparison of microbiological characteristic among clinical isolates of community-acquired (CA), healthcare-associated (HCA), and nosocomial *K. pneumoniae* bacteremia

Microbiological characteristics of isolates	CA (*n* = 70)	HCA (*n* = 102)	Nosocomial (*n* = 165)	*p* value		
				CA vs. HCA	HCA vs. Nosocomial	CA vs. Nosocomial
Capsular type K1	12 (17.1)	10 (9.8)	8 (4.8)	0.157	0.117	0.002
Capsular type K2	13 (18.6)	12 (11.8)	9 (5.5)	0.213	0.063	0.002
Capsular type K1 and K2	25 (35.7)	22 (21.6)	17 (10.3)	0.041	0.011	< 0.001
Capsular type K1, K2, K5, K20, K54, and K57	36 (51.4)	37 (36.3)	32 (19.4)	0.048	0.002	< 0.001
Isolates with both antimicrobial resistance^a^ and capsular type K1, K2, K5, K20, K54, and K57	2 (2.9)	6 (5.9)	9 (5.5)	0.475	0.883	0.513
Isolated with both multidrug resistance^b^ and capsular type K1, K2, K5, K20, K54, and K57	2 (2.9)	3 (2.9)	7 (4.2)	1.000	0.746	1.000
Presence of plasmid *rmpA*	42 (60.0)	39 (38.2)	36 (21.8)	0.005	0.004	< 0.001
Presence of plasmid *rmpA2*	41 (58.6)	37 (36.3)	35 (21.2)	0.004	0.007	< 0.001

^a^Antimicrobial resistance is defined as non-susceptibility to at least one antimicrobial agent in addition to ampicillin

^b^Multidrug resistance is defined as non-susceptibility to at least one agent in three or more antimicrobial categories

## Discussion

The mortality of HCA *K. pneumoniae* bacteremia was comparable to that of CA bacteremia but significantly lower than that of nosocomial bacteremia. The rate of MDR phenotype, virulent capsular types and *rmpA/rmpA2* genes in the isolates from HCA bacteremia was between those from CA and nosocomial bacteremia. The rate of isolates with virulent capsular types in HCA infection was comparable to that in CA infection. However, the rate of MDR phenotype in HCA infection was higher than that in CA infection. Our findings support that HCA bacteremia is a category distinct from CA and nosocomial bacteremia in terms of the clinical and microbiological features, which was addressed in the previous study [12].

The clinical characteristics of CA, HCA, and nosocomial *K. pneumoniae* bacteremia have rarely been compared [2]. Meatherall et al. have found that among the 640 cases of *K. pneumoniae* bacteremia in Canada, 43, 30, and 27% were HCA, CA, and nosocomial infections, respectively [2]. Meatherall et al. only compared the primary source of infection among the three groups. The comparison of antimicrobial resistance and capsular types among the three groups were lacking. In the current study, we used similar definition to define HCA bacteremia, and 30.3% were classified as HCA infection. With regard to the foci of infection, the rates of liver abscess were 2.3% among all patients with bacteremia in Canada [2], and 6.2% among all patients with bacteremia in our study. Moreover, it was higher in the CA group (20.0%) than in the other two groups (4.9% in the HCA group, 1.2% in the nosocomial group). *K. pneumoniae* isolates with virulent capsular types responsible for liver abscess was more common in East Asia than western countries. Our results underscored the endemic nature of *K. pneumoniae* liver abscess in Taiwan [9, 21, 22].

Our previous study conducted from 2007 to 2010 showed that patients with HCA *K. pneumoniae* bacteremia had a higher infection-related mortality than CA *K. pneumoniae* bacteremia (31.7% versus 13.5%, *p* < 0.001) [13]. In a study conducted in Korea from 2003 to 2008, the 30-day mortality rate of HCA *K. pneumoniae* bacteremia was higher than that of CA *K. pneumoniae* bacteremia (22.4% versus 11.3%, *p* = 0.001) [14]. However, HCA bacteremia was not associated with a significantly higher 28-day mortality than CA bacteremia in the current study (17.6% versus 14.3%, *p* = 0.557). HCA bacteremia is rising in prominence and physicians are becoming more familiar with this category, which may contribute to a lower threshold for investigation and detection bias resulting in improved prognosis. Furthermore, the mortality of patients with HCA bacteremia was significantly lower than those with nosocomial bacteremia. In the literature, nosocomial *K. pneumoniae* bacteremia is usually associated with a higher mortality than community-onset *K. pneumoniae* bacteremia [4]. Our study re-emphasized the severity and grave prognosis of nosocomial *K. pneumoniae* bacteremia.

In the present study, nosocomial isolates are still notorious for the highest rate of resistance (52.7%). The proportion of CA isolates that did not display wild-type susceptibility (14.2%) was similar to that (14.9%) in our previous study conducted from 2007 to 2010 [13]. However, HCA isolates also had a higher rate of antimicrobial resistance that is indicated by non-wild-type susceptibility (34.3%) than HCA isolates (20.7%) in our previous study [13]. It might be caused by the increased exposure to antimicrobials in the hospital environments that would promote the selection pressure for antimicrobial resistance. It may also suggest that more drug-resistant *K. pneumoniae* have spread to the community or the wild-type strains have evolved and became resistant. The characterization of HCA bacteremia isolates might be helpful in choosing an empirical antimicrobial therapy, as supported by the original intention. Moreover, the surveillance of the resistance pattern of HCA isolates can help in monitoring the development of drug-resistant *K. pneumoniae* in the community.

Tsay et al. have previously compared the serotypes of community-onset and nosocomial *K. pneumoniae* bacteremia strains [4]. The serotype K1 strain was more frequently observed in community-onset infections than in nosocomial infections (29.7% versus 14.0%, *p* = 0.02), and the non-typeable strains were more prevalent in nosocomial infections than in community-onset infections (75.0% versus 55.3%, *p* = 0.01) [4]. Cubero et al. have recently investigated the molecular epidemiology of CA, HCA, and nosocomial *K. pneumoniae* bacteremia via multi-locus sequence typing and pulsed-field gel electrophoresis in Spain [23]. However, data on the capsular types were lacking. This study first showed that CA *K. pneumoniae* isolates had the highest rate of virulent capsular types (K1, K2, K5, K20, K54, and K57), followed by the HCA isolates, whereas the nosocomial isolates had the lowest rate of virulent capsular types among the three categories. The distribution of *K. pneumoniae* isolates with *rmpA/rmpA2* genes among CA, HCA, and nosocomial bacteremia was similar to that of the virulent capsular types. Classic *K. pneumoniae* and hypervirulent *K. pneumoniae* strains were proposed as the two different pathotypes of *K. pneumoniae* recently [24]. Hypervirulent *K. pneumoniae* were primarily responsible for community-onset pyogenic infections in Taiwan, and classic *K. pneumoniae* with antimicrobial resistance usually involved in nosocomial infections. The presence of *rmpA*/*rmpA2* genes and virulent capsular types were usually associated with hypervirulent strains. The current findings support the unique microbiological features between nosocomial versus CA strains. Notably, approximately 19.4% of the nosocomial strains belonged to these virulent capsular types. This is of concern that these virulent strains are spreading and no longer restricted in the community. The virulent strains in hospitals must be monitored to prevent fatal outcomes among susceptible patients. The occurrence of virulent strains in HCA bacteremia might indicate the transmission between community and hospital. The active molecular screening of capsular genotypes and *rmpA* in *K. pneumoniae* isolates in hospitals might be an effective strategy in controlling and preventing the spread of infection.

The present study had several limitations. First, incomplete medical records excluded a certain proportion of the total population that should have been included. However, only 2 patients were excluded accounting for very low proportion (0.6%) of the total population. Second, the absence of evidence pertaining to prior antibiotic exposure does not equate to evidence of absence, especially in the CA and HCA *K. pneumoniae* bacteremia patients. Third, the difference of mortality rates among the three categories might be related to confounding variables that differed significantly and multivariate analysis was not performed to investigate mortality rates further. Fourth, we did not perform multilocus sequence typing of *K. pneumoniae* isolates to delineate the clonal distribution and genetic diversity among CA, HCA, and nosocomial bacteremia. Finally, our study was conducted in a single tertiary-care teaching hospital where the patient populations, clinical practice, and treatment outcome may differ from those in other non-tertiary-care hospitals. Thus, the results may not be generalizable.

## Conclusions

HCA *K. pneumoniae* bacteremia is a category distinct from CA and nosocomial infections in terms of clinical and microbiological features. The characterization of the clinical characteristics of CA, HCA, and nosocomial bacteremia will help professionals to better manage patients. Further studies on the microbiological characteristics of HCA strains must be conducted to accurately identify the transmission of virulent or antimicrobial-resistant strains between community and hospital. We also encourage further studies from different geographical locations to assess the global distribution and trends in this research topic.

## Abbreviations

APACHE: Acute physiology and chronic and prevention evaluation
CA: Community-acquired
HCA: Healthcare-associated
MDR: Multidrug-resistant
SPSS: Statistical package for the social sciences
